# Applying 3D cultures and high-throughput technologies to study host-pathogen interactions

**DOI:** 10.3389/fimmu.2025.1488699

**Published:** 2025-02-20

**Authors:** Elaine Cristina Pereira De Martinis, Virgínia Farias Alves, Marita Gimenez Pereira, Leonardo Neves Andrade, Nathália Abichabki, Anna Abramova, Mirjam Dannborg, Johan Bengtsson-Palme

**Affiliations:** ^1^ Ribeirão Preto School of Pharmaceutical Sciences, University of São Paulo, Ribeirão Preto, São Paulo, Brazil; ^2^ Faculdade de Farmácia, Universidade Federal de Goiás, Goiânia, Goiás, Brazil; ^3^ Division of Systems and Synthetic Biology, Department of Life Sciences, SciLifeLab, Chalmers University of Technology, Gothenburg, Sweden; ^4^ Centre for Antibiotic Resistance Research (CARe), Gothenburg, Sweden; ^5^ Department of Infectious Diseases, Institute of Biomedicine, University of Gothenburg, Gothenburg, Sweden

**Keywords:** tridimensional cell culture, organoids, organ-on-a-chip, rotating wall vessel (RWV), high-throughput sequencing, transposon sequencing (TnSeq), microbial model communities

## Abstract

Recent advances in cell culturing and DNA sequencing have dramatically altered the field of human microbiome research. Three-dimensional (3D) cell culture is an important tool in cell biology, in cancer research, and for studying host-microbe interactions, as it mimics the *in vivo* characteristics of the host environment in an *in vitro* system, providing reliable and reproducible models. This work provides an overview of the main 3D culture techniques applied to study interactions between host cells and pathogenic microorganisms, how these systems can be integrated with high-throughput molecular methods, and how multi-species model systems may pave the way forward to pinpoint interactions among host, beneficial microbes and pathogens.

## 1 Introduction

The study of microbe-microbe interactions as well as microbial interactions with their hosts represents a grand challenge for science. The advancement of high throughput sequencing technologies has provided valuable tools to better understand microbial diversity and function in the human microbiome, especially when combined with the culturing of fastidious pathogens and members of human microbiota (1).

Animal-based models have played a critical role over the years for research on the microbiome, as well as in several other fields related to biological and biomedical sciences, since some animals – such as rodents – present a conserved common structural organization that allows the modeling of human conditions (2, 3). However, in recent years there has been increasing opposition to the excessive use of animals in experiments, especially for ethical reasons, which has led to a search for robust alternative models, such as *in vitro*, *ex vivo*, and *in silico* methods that can be used in scientific research as adjuncts to or in replacement of animal models (4).

Eukaryotic cell culture is an interesting alternative to animal models for many biomedical applications, but these methods are limited because they usually involve growing cell lines in monolayers, failing to mimic important tissue functions. To improve these models, cell lines can be grown in three-dimensional culture (3D), hence developing some of the typical tissue structures, such as expression of tight junction proteins and production of mucin in the case of intestinal cells (3, 5, 6). Moreover, different types of cell lines can be grown in 3D cultures, as outlined in **Table 1**, but their advantages and drawbacks must be taken into account to allow the best choice of model for each application.

**Table 1:** Types of eukaryotic cells that can be used in three-dimensional models.

Type of cells for culture	Advantages	Disadvantages and remarks
**Immortalized cell lines**	Multiple batches obtained at low cost, easy to expand and to handle from frozen stocks. Adequate for high-throughput screening of virulence factors. Facilitate the understanding of specific cellular mechanisms. Standardization of materials and methods. Unlimited supply of materials. Less ethical concerns. Unlimited growth potential.	As they are frequently cancerous, transformed or genetically immortalized, they fail to mimic important tissue functions (e.g. polarization, barrier formation, differentiation). Serial passages can lead to genotypic and phenotypic variations.
**Stem cells and explants**	Because they are isolated directly from a specific, untransformed tissue, they closely mimic tissue functions *in vitro*, increasing their biological significance. Can be used in advanced cell culture models to optimize and refine experimental conditions. Can originate from healthy and sick donors, which allows the study of cells with diverse characteristics. Can be isolated from frozen tissue, enabling studies on organoids long after tissue samples were obtained.	Single batch obtained from a donor. Dependent on the availability of fresh tissue. Slower and more limited growth, limited expansion potential. High cost (although cheaper than using laboratory animals). The characteristics of primary cell lines can change with each subsequent passage. Since they are taken from different donors, there may be heterogeneity of behavior even when faced with the same stimuli. Absence of metabolic regulatory mechanisms that exists *in vivo*. Culture conditions need to be optimized. While organoids can be grown from frozen and thawed tissues, the freezing process affects the basal metabolic rate of the cells. Require authorization for use.

Sources: Based on (7–9).

Three-dimensional cell culture has been applied in developmental, cellular, and cancer biology, as well as for studies of host-bacterial interactions since it mimics important characteristics that occur *in vivo*, including cell-cell and cell-extracellular matrix interactions in an *in vitro* system (6, 10, 11). Such 3D cultures represent a middle ground between monoculture experiments and animal models for the study of infectious diseases, especially if combined with high-throughput technologies. This combination aids in determination of host-specific immune responses and pathogen interactions, leading to new insights into both the pathogenesis and treatment of infections (12–14).

In view of the increasing accessibility and affordability of high-throughput techniques (e.g. transcriptomics, proteomics, and metabolomics), there are great opportunities for measuring the responses of 3D cultures in model systems at a very large scale, both on the eukaryotic tissue side and on the side of bacteria interacting with the host (14, 15). Thereby, experiments with 3D culture can improve the understanding of the host interactions with different microbial strains, as well as various drugs and chemicals.

To further increase the relevance of co-culture systems involving interactions between 3D-cultured human cells and bacteria one can introduce microbial communities rather than single species, to test for interactions in a multi-species context. However, as microbial communities are often highly complex and contain hundreds or thousands of species, the results of this approach are often hard to mechanistically interpret. To gain a better understanding of the mechanisms behind different interactions, microbial model communities can be used (16). Such microbial model communities offer a tradeoff allowing a reasonable degree of community complexity, but at the same time permit mapping a large part of the interactions between the microbes in the communities (and their host), which makes it possible to describe how the interactions are mediated and what genes are responsible for community behaviors.

Considering the importance of 3D eukaryotic cell culture models for studying host-microbe interactions, the main advantages and drawbacks of different systems available will be discussed in the next sections. Finally, the latest developments in 3D culture techniques combined with high-throughput molecular methods and multi-species model systems will be presented in the light of possible interactions among the host, beneficial microbes and pathogens.

## 2 Three-dimensional models for eukaryotic cell culture

The most recent advances in the development of technologies for 3D cultures of eukaryotic cells include multicellular spheroids, organoids, hydrogels, organ-on-a-chip platforms, and 3D bioimprints. Different principles and protocols for 3D cultures are used to recapitulate the morphology, as well as the functionality and microenvironmental characteristics of human tissues and organs, with different methods providing more realistic or reliable study systems for various purposes (**Table 2**). In particular for research on pathogenic bioagents, rotating wall vessel (RWV) bioreactors, extracellular matrix (ECM)-embedded/organoid models, and organ-on-a-chip (OAC) models have been used (15). RWV models have advanced to incorporate immune cells, allowing their role in host-microbe interactions to be elucidated (15). However, the choice of system depends on various factors, such as cost, technical complexity, and expertise needed to run experiments with the model.

**Table 2:** Strengths, weaknesses, and applications of different 3D culture systems.

3D culture system	Main advantages	Main disadvantages	Potential applications	Main references
Spheroids				
Spherical 3D cellular aggregates, self-assembled in a nonadherent environment.	Allows study of the regulation of multiple genes expressed in the tissue-like structures (e.g., stress response, inflammation, etc).	Formation of gradient and lack of nutrients in the spheroids’ cores, especially with larger sizes.	Studies on gastrointestinal development, mucosal immunology and epithelial infection.	(17–20).
Organoids				
3D self-organized arrangements derived from untransformed primary cells. Can be generated from two types of stem cells: *(i)* pluripotent stem cells (embryonic or induced), or *(ii)* organ-specific adult stem cells (tissue-specific resident stem cells).	Pluripotent stem cells can differentiate into various cell types and allow for large-scale experiments both *in vitro* and *in vivo*. Organ-specific adult stem cells can be obtained from patients with diverse genetic and disease backgrounds and allow to study the onset and progression of diseases. Organoids from organ-specific adult stem cells present potential for expansion with very high genetic stability and tissue-like maturity.	Organoids derived from pluripotent stem cells harbor different cell lineages, such as epithelial and mesenchymal cells. Organoids from adult stem cells have limited self-renewal capacity for long-term applications, and are less suitable for gene knockout and precise gene-editing techniques in comparison with pluripotent stem cells.	Organoids based on stem cells are very useful to study cancer biology, diseases linked to dysbiosis (e.g. Inflammatory Bowel Disease) and various infectious diseases.	(8, 21–23).
Rotating-Wall Vessel bioreactors				
Eukaryotic cell lineages are cultivated with collagen beads in a microgravity environment leading to spontaneous development of 3D tissue-like structures.	The system is flexible and can be adapted to different types of cells and assays. For intestinal cells, the apical cellular region of the tissue is located on the opposite side of the microcarrier bead, offering advantages for studying host-gut microbes allowing direct contact between test microorganisms and the apical portion of epithelial cells.	Time to optimize culture conditions for aggregate formation, cellular differentiation and characterization for each new model system and each new cell type.	3D aggregates can be used for structural analysis (e.g. microscopy, evaluation of cell signaling, gene expression, toxicity assays and studies of host-pathogen interactions.	(24, 25).
Hydrogel Scaffolds				
Formed by networks of water-soluble polymers crosslinked through covalent bonds or joined by physical attraction forces.	Replicate the complexity of living tissues well (cell attachment, growth and differentiation). Different techniques available to set up the system (e.g., bioprinting and electrospinning).	Mechanical properties have to be optimized to achieve the desired cell density, distribution and material-cell interactions.	Studies of host-pathogen interactions, cell transplantation, controlled drug delivery and wound healing.	(26–28).
Organs-on-chips platforms				
Biomimetic platforms combining biomaterial technology, microfluidics and tissue engineering to create miniature tissue devices.	Models exist for mimicking gut, skin, kidney, lung, heart, liver, blood vessels and bone marrow. Multiple organs can be connected for the study of different organs at the same time. Possible use for high throughput screening when integrated with automation smart analysis systems.	The technology is still under development and the models cannot perform all the functions of an organ. The systems need expertise in multiple areas and is not easy to use in non-specialized laboratories.	Elucidation of the pathophysiology of infectious agents in different body sites and studies to advance personalized medicine, drug delivery and therapeutics.	(29–31).

### 2.1 Spheroids

**Figure 1:**
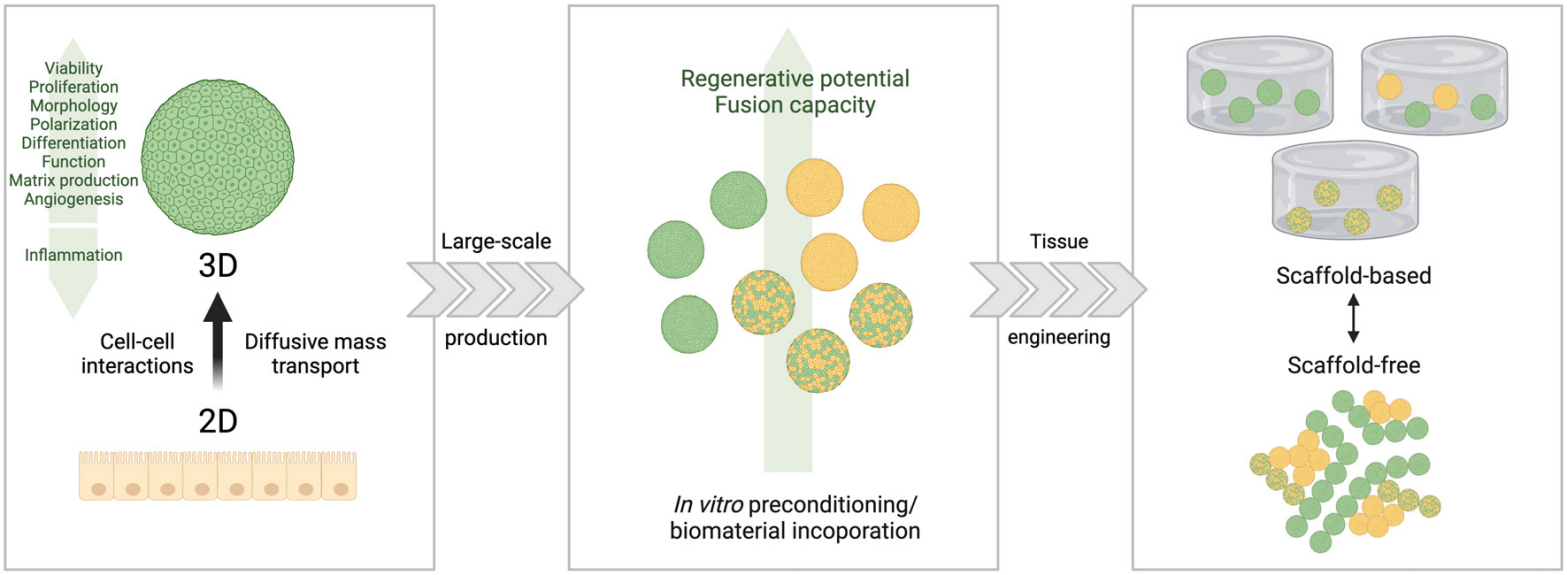
Scheme outlining the main steps and features in spheroid formation. Spheroids (3D) are derived from monolayer cultures (2D) but present higher degrees of cell-cell interaction and diffusion of fluids, leading to the expression of tissue-like properties (such as higher viability, proliferation and morphology, combined with less inflammation). Spheroids are adequate for large scale production by combining biomaterials with the cells, which can be used in systems with or without scaffolds, for different applications. Based on Laschke and Menger (32).

Spheroids are spherical 3D cellular aggregates, self-assembled in a nonadherent environment. The formation of spheroids occurs initially by the loose agglomeration of single cells from culture in suspension in a process of self-assembly. This is followed by the strong adhesion of initial cell aggregates promoted by homophilic binding between cadherins of peripheral cells, signal transduction by β-catenin and increased contact between adjacent cells promoted by actin, forming strong adhesive multi-cellular structures (20). There are different techniques for culturing spheroids, including pellet culture, liquid overlay, hanging drop, spinner culture, RWV, microfluidics, and magnetic levitation (20). Spheroids have positive regulation of multiple genes associated with the response to stress, inflammation, redox signaling, hypoxia, and angiogenesis (17, 18). This is advantageous for studying gastrointestinal development, mucosal immunology, and epithelial infection (19). However, the main disadvantage of this model is the formation of a diffusion gradient that occurs with increased spheroid size and can cause a lack of nutrients in the core of the spheroid (20). Some possible applications of spheroids include tumor models, tissue engineering, transplantation therapy, as well as drug screening (2, 32, 33). **Figure 1** illustrates the steps of spheroid formation.

Some studies have reported on host-pathogen interactions using spheroids of eukaryotic cells. For example, Osswald et al. (34) observed that *Salmonella enterica* Typhimurium YB1 selectively localized, survived, and replicated in hypoxic areas within multicellular tumor spheroids (MCTS) from the HT-29 cell line. Furthermore, those authors reported that spores of *Clostridium* sp*orogenes*, a strict anaerobe, germinated in the MCTS hypoxic areas. Such results indicate that spheroids can be an appropriate and reliable model to investigate live bacteria in their interactions with host tissues. In another study, Mukundan et al. (35) produced spheroids from THP-1 human monocyte/macrophage cells infected with mCherry-expressing *Mycobacterium bovis* BCG (Bacilli Camille Guérin) to mimic *Mycobacterium tuberculosis* infection and to create an HIV-TB (Human Immunodeficiency Virus - tuberculosis) co-infection model. In that study, the authors concluded that the spheroid system was useful for monitoring the kinetics of BCG growth, for studying HIV-TB co-infection and for tracking the anti-TB response of potential drug candidates. Broadly speaking, recent studies indicate spheroids prepared with eukaryotic cells are useful tools that deserve to be further explored to elucidate different mechanisms of host-microbe interactions.

### 2.2 Organoids

**Figure 2:**
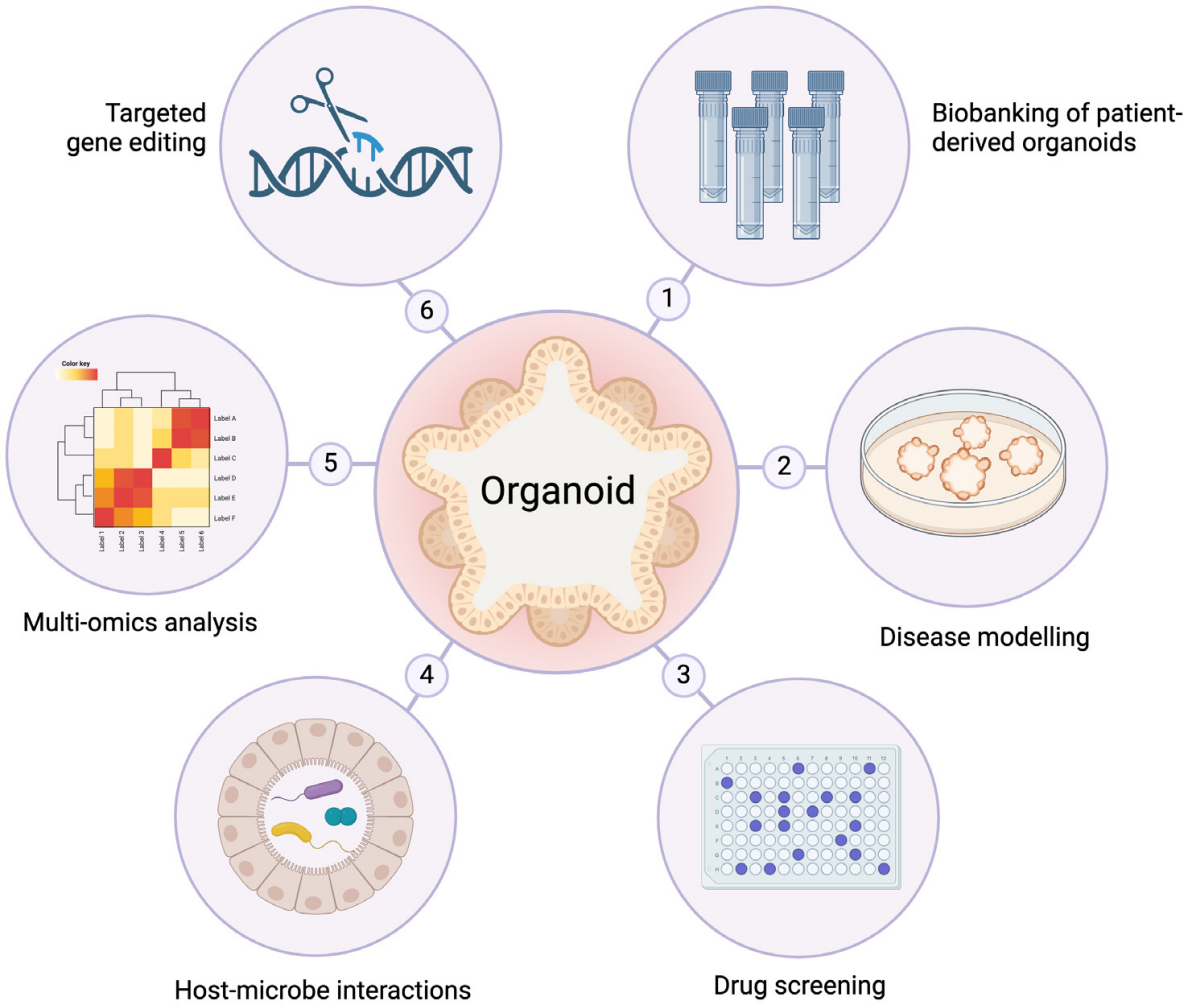
Schematic representation of organoid applications. 1 - Biobanking: organoids can be prepared from tissue resections after surgery and kept frozen in liquid nitrogen, which is important for studies on, e.g., personalized cancer therapy. 2 - Disease modelling: differentiated cells can be derived from human pluripotent stem cells and used for xenotransplantation in murines, serving as model phenotyping tool. 3 - Drug screening: organoids are appropriate for efficacy and toxicity testing. 4 - Host-microbe interactions: organoids are ideal models that recapitulate *in vivo* conditions for studies using human brain tissues (Zika virus) and intestinal cells (norovirus and rotavirus), among other infectious diseases. 5 - Multi-omics analysis: high-throughput characterization of healthy or diseased organoids can be done with regards to transcripts, proteins, metabolites, etc. 6 - Targeted gene editing: sequential mutations can be introduced into normal organoids to mimic different patient tumor formation scenarios (e.g., studies on colorectal carcinomas using CRISPR/Cas9). Figure based on Dutta et al. (39) and Hohwieler et al. (41).

Organoids consist of 3D self-organized arrangements derived from untransformed primary cells, either Human Pluripotent Stem Cells (HPSC) or adult stem cells. These cells present the capacity to differentiate into various cell types, resembling natural properties of *in vivo* tissues, including protein expression, nutrient absorption, and barrier function (8, 36, 37). In organoid cultures, isolated stem cells receive, under specific culture conditions, combinations of selected growth factors to induce cell differentiation, so that the 3D structural organization, multicellularity, and functions of the *in vivo* target tissue/organ of interest are mimicked, hence they are also referred to as “organ in a dish” systems (8, 38–40). It is possible to produce organoids from virtually all human tissues, although brain and cardiac organoids can be only HPSC-derived, enabling the connection of cell culture and large-scale experiments in a variety of *in vivo* settings (21, 22) (**Figure 2**).

An organoid generated from induced HPSC can harbor different cell lineages, such as epithelial and mesenchymal cells (8). Moreover, HPSC-derived organoids can be expanded to enable large-scale studies, such as drug screening and metabolic profiling (22). Organoids generated from adult tissue-specific stem cells consist of pure epithelial cells and generally present potential for expansion with very high genetic stability and tissue-like maturity (8, 42). However, these organoids have limited self-renewal capacity for long-term applications and are less suitable for gene knockout and precise gene-editing techniques, in comparison with HPSC-derived organoids (22). On the other hand, adult organoids can be derived from stem cells from a single individual, allowing the formation of an isogenic tissue with identical genes (42). Furthermore, adult organoids derived from different donors open up the possibility of developing induced pluripotent stem cells from patients with diverse genetic and disease backgrounds, which can leverage studies for understanding the onset and progression of those diseases, as well as potential treatments (22). This kind of approach, taking into consideration diverse genetic backgrounds, may help the study of diseases linked to dysbiosis, such as Inflammatory Bowel Disease (43, 44).

Organoids can be co-cultured (microinjected) with microorganisms to further simulate the tissue niche, thus constituting excellent platforms to evaluate host-pathogen dynamics, as well as to assist in the development of effective control measures against pathogens. This is especially true because organoids mimic the natural site of interaction, the natural target cell of infection, as well as the host factors that contribute to the outcome of the infection (45). Although there are model limitations, such as deficiency of immune cells, vasculature and innervation, the use of organoid models brings future perspectives to study not only known infectious diseases, but also future emerging ones (46).

Respiratory organoids, such as lung alveolar, lung airway, and bronchial organoids, have been used as models for infections caused by a variety of agents, including bacteria, fungi, parasites, and viruses, providing valuable insights into the underlying host-pathogen interactions at the cellular and molecular levels (45, 47). The recent COVID-19 (coronavirus disease 19) pandemic, caused by the SARS-CoV-2 virus, greatly boosted the development of new human lung organoid models. These models have contributed to better understanding the physiopathology of the disease with regards to host response and cellular damage mediated by immune cells, as well as the identification of potential therapeutic targets and the discovery of novel drugs (22, 46).

Human brain organoids (HBOs) have provided new physiologically relevant systems for functional modeling of the brain, as they can mimic the dynamic spatiotemporal process of early brain development. Indeed, HBOs have been used to study infectious agents with brain tropism, such as Zika virus (ZIKV), herpes simplex virus (HSV-1), human cytomegalovirus (HCMV) and *Toxoplasma gondii* (48). Watanabe et al. (49) developed an HBO system similar to the human fetal brain *in vivo*, efficiently reproducing cortical and basal ganglia structures, with functional neurons exhibiting spontaneous network-like activities, as demonstrated by immunohistochemical, transcriptomic, and electrophysiological analyses. Those authors were able to model the teratogenic effects of the ZIKV in the developing brain, identifying susceptibility receptors and therapeutic compounds that could mitigate the destructive action of ZIKV. Krenn et al. (48) used HBOs to reproduce the microcephaly-like phenotype caused by ZIKV and HSV-1, two viruses with different structures, sizes and modes of replication (48). The authors observed that both viruses replicated efficiently in the HBO system, causing features of microcephaly, albeit with major differences in the underlying structural defects and transcriptional profiles, as well as differences in involvement of the antiviral system and sensitivity to IFN-I (interferon -1).

Human intestinal organoids (HIOs) have been used to study enteric viruses and bacterial infections, as well as to evaluate commensal intestinal microbes (42, 50). HIOs derived from induced HPSCs, in addition to the luminal cells, present the majority of other cell types present in the human intestine, mimicking the *in vivo* native tissue architecture with villus and crypt domains, providing an effective system for studying the intestinal epithelium and its interaction with various stimuli, including enteric pathogens (38, 51). Using HIOs, Engevik et al. (52) demonstrated that *Clostridioides difficile*, a major cause of antibiotic-induced diarrhea, showed a decreased expression of the core protein MUC2 that is secreted by Goblet cells, affecting the thick layer of mucus that acts as a physical barrier against pathogens (52). On the other hand, Schulte et al. (53) developed an HIO model that comprised epithelial and endothelial cell layers, as well as a collagen matrix and immune cells, to study the acute phase of *S.* Typhimurium infection. The model mimicked human gastroenteritis by restricting the pathogen to the epithelial compartment, providing an advantage over existing mouse models (53). Intestinal organoids derived directly from adult tissues and made up only of epithelial cells are called enteroids or colonoids, and provide a physiologically relevant platform that, in addition to allowing the investigation of altered intestinal function and drug efficacy, also enables evaluation of interactions with microbes (6, 54). Using human small intestinal explants and enteroids, Sheikh et al. (55) demonstrated that EatA, a secreted autotransporter protein produced by some Enterotoxigenic *Escherichia coli* (ETEC) strains, plays a crucial role in the development of infection by degrading the mucin barrier, promoting microbial access to enterocytes, followed by toxin release. Enteroid systems have been used to clarify the pathogenicity mechanisms of infections caused by viruses (e.g. Norovirus, Adenovirus, Rotavirus) and bacteria (e.g. *Salmonella*, ETEC, enterohemorrhagic *E. coli* - EHEC) in the gastrointestinal tract (51, 55, 56).

### 2.3 Rotating-Wall Vessel bioreactors

Insights into microbe-host interactions can also be obtained with the organotypic Rotating-Wall Vessel (RWV) bioreactors, also known as “microgravity” reactors, which provide physiological-like conditions of fluid-shear, level of oxygenation and nutrients, favoring the development of specialized features of *in vivo* tissues, including spontaneous differentiation into multiple epithelial cell types, polarization, and cell-cell interactions (15, 25). In the RWV bioreactor, cells are cultivated with collagen-coated spheres or microcarrier matrices, that allow the spontaneous development of cohesive 3D structures representative of the parental tissue (24, 25). An interesting feature of RWV-bioreactors platforms for the study of host-microbe interactions is that the apical cellular region of the epithelial tissue is located on the opposite side of the collagen coating, allowing a better mimicry of *in vivo* conditions, since the microorganisms directly encounter the apical portion of the epithelial cells (24).

RWV-derived intestinal models have been used for studying interactions between eukaryotic cells and a variety of pathogens, such as *S.* Typhimurium (including multidrug-resistant strains from ST313), *S.* Typhi, pathogenic *E. coli* (EPEC, EHEC), *Cryptosporidium parvum*, *Listeria monocytogenes*, and human enteroviruses, including coxsackievirus B and poliovirus (5, 15, 24, 57–64). For example, Barrila et al. (64) developed and validated a novel RWV-based 3D co-culture infection model for *Salmonell*a, using colonic epithelial cells and macrophages. For that, the authors first activated the pro-monocytic cell line U937 on collagen-coated scaffolds, and next added the HT-29 epithelial cells to the RWV 3D model for culturing until optimal differentiation was achieved. Those authors then used different *Salmonella* strains for studies of host-pathogen interactions and observed pathovar-specific differences in colonization patterns and intracellular colocalization. Overall, that study highlighted the usefulness of the RWV 3D model for identifying microenvironmental factors important for regulating infection.

Carvalho et al. (5) studied the interactions of EPEC and EHEC with human intestinal cells grown in microgravity in a rotary 3D cell culture system. As in many studies, this study did not directly compare how the bacteria adapted to organoids versus monolayers, nor whether the organoids showed a more natural response to bacterial exposure than monolayer cells. That said, the authors did find that organoids expressed more normal markers of intestinal tissue than conventionally grown monolayers, although these experiments were performed in the absence of bacteria. Organoids also produced higher levels of intestinally expressed disaccharidases and alkaline phosphatase than monolayers. In addition, the organoid HCT-8 developed microvilli and desmosomes characteristic of normal tissue (5). This speaks in favor of the organoids being more representative and more differentiated than HCT-8 cells grown as monolayers, which also implies that this would be a more realistic host-pathogen model.

Besides being a good model for the study of host-pathogen interplay, RWV bioreactors can be useful in assessing possible interactions between beneficial microbes and eukaryotic cells. For example, Pereira et al. (24) used the RWV microgravity model to study interactions between *Lactobacillus sakei* 1 (a potential probiotic strain) and Caco-2 cells, carrying out assays of adhesion/invasion and transcriptomics to elucidate the genes that were up or downregulated in intestinal cells by the presence of the lactic acid bacterium.

### 2.4 Hydrogel scaffolds

**Figure 3:**
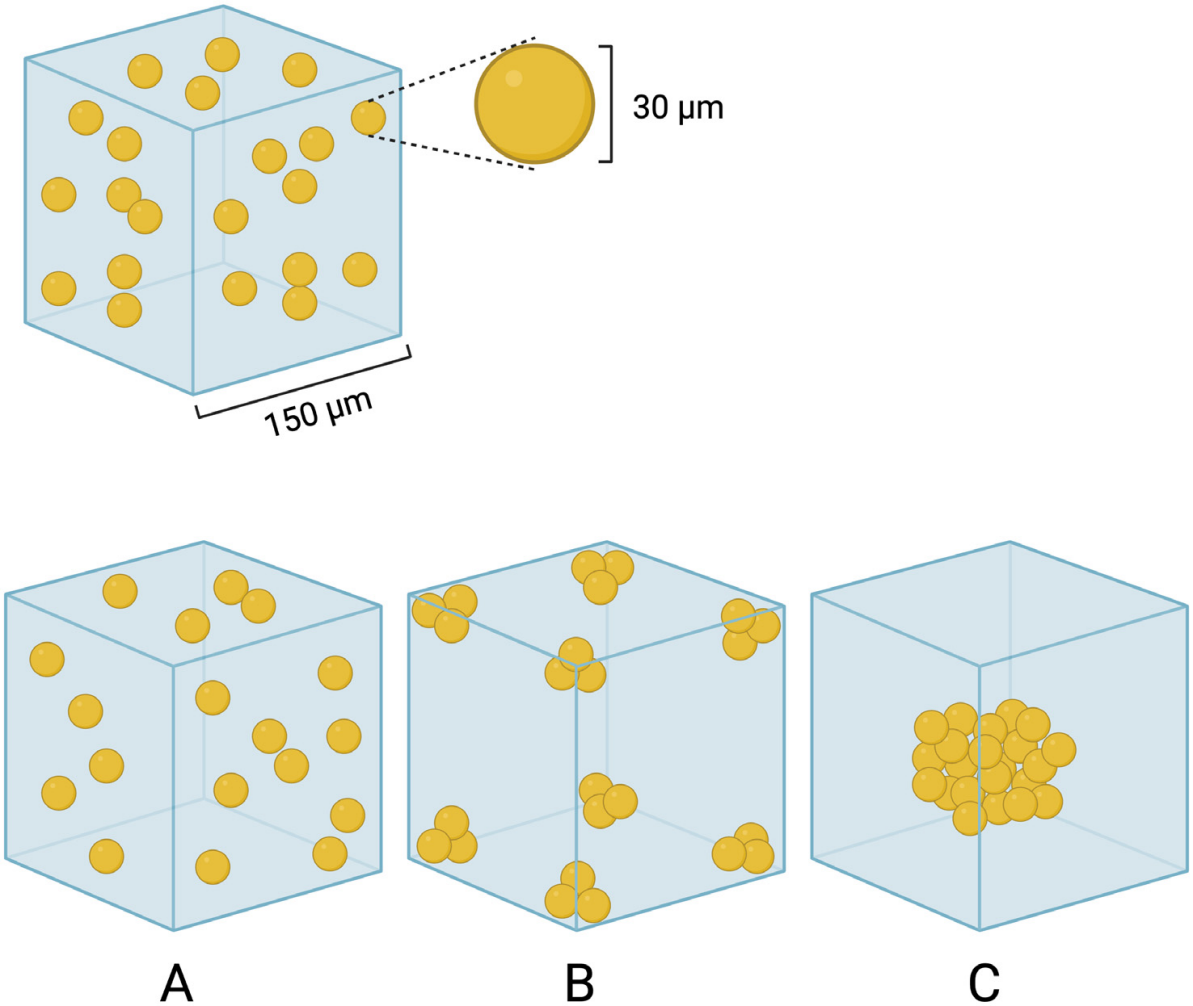
Simplified representative hydrogel models in bioprinting, with possible cell distribution within the matrix: **(A)** random, **(B)** eight corner clusters and **(C)** central cluster. Based on Hölzl et al. (27), with permission under a Creative Commons license (https://creativecommons.org/licenses/by/3.0/).

Hydrogels are 3D networks of water-soluble polymers from natural or synthetic sources, crosslinked either through covalent bonds or joined by physical intra- and intermolecular attraction forces (26). Hydrogels made with permeable biocompatible materials have been used as scaffolds to replicate the structural complexity of living tissues, as they provide a microenvironment for cell attachment, growth, differentiation, and interactions (10, 65). Hydrogel scaffolds can be obtained by several techniques, such as bioprinting and electrospinning (26). Bioprinting is a process based on additive manufacturing from materials containing living cells. These materials (so called bioink) are based on cytocompatible hydrogel precursor formulations and their properties are essential for printability: structural resolution, shape fidelity, and cell survival. **Figure 3** illustrates possible cell distributions obtained in bioprinting, based on a study by Hölz et al. (27), for modeling the influence of cell density and mechanical properties of matrices (shear and loss modulus) during hydrogel fabrication. According to Hölz et al. (27) higher cell density can result in reduced modulus of hydrogels. Those authors also observed that hydrogel samples with edge and central cell clusters were softer compared to the ones with randomly distributed cells, but interestingly cluster distribution had minimal impact on the accumulated cell loadings in general.

Overall, models based on numerical approaches are very useful for depicting the cellular mechanics within hydrogels, allowing for the prediction of mechanical properties to achieve the desired cell density, distribution, and material–cell interaction, resulting in a suitable matured bioprinted tissue construct.

Electrospinning of hydrogels can also be used to produce scaffolds (26). In short, electrospinning is a process that uses electrostatic forces to pull fibers from a polymer solution and create nanofibrous structures that mimic the characteristics of extracellular matrices, providing a natural environment for tissue formation (66, 67). This versatile technique has relatively low experimental complexity and allows the use of a wide variety of biocompatible and biodegradable polymers to produce electrospun nanofiber scaffolds with physicochemical properties that promote cell adhesion, proliferation, and differentiation (26, 66, 67).

Hydrogel scaffolds have recently been applied to mimic host-pathogen interactions. In a study by Huang et al. (68), a mucus-like hydrogel was prepared using alginate-mucin (ALG-MUC) polymers crosslinked with calcium chloride. The hydrogel was incorporated into an aqueous two-phase system co-culture platform containing polyethylene glycol and dextran, to simultaneously support the growth of a monolayer of mammalian cells (16-HBE or Caco-2) and pathogenic bacteria (respectively for experiments with *Pseudomonas aeruginosa* or *Shigella flexneri*). The authors argued the ALG-MUC hydrogel combined with the polymer-based liquid microbial scaffold was suitable for mimicking the complexity of the human microbiome niche (68).

Alzheimer et al. (12) proposed to use an extracellular matrix structure named Small Intestine Submucosa – “SISmuc” – to study infection by *Campylobacter jejuni* in Caco-2 cells. That hydrogel scaffold allowed the formation of a polarized epithelial barrier and to follow the infectious process. Furthermore, the 3D platform enabled the identification of isolate-specific infection phenotypes, as well as relevant outcomes of the infectious process. Moreover, it was revealed that a small RNA pair was involved in the pathogenesis of *C. jejuni*, which suggested “SISmuc” could be useful for studying gastrointestinal pathogen infection mechanisms (12).

Biomimetic bone scaffolds containing collagen and minerals can also be useful for studying other host-pathogen interactions, such as in the case of osteomyelitis – an inflammatory disease of the bone caused by a wide range of opportunistic pathogens. Parente et al. (13) developed an osteomyelitis model based on murine osteoblastic cells (MC3T3-E1) to study infection by *Staphylococcus aureus*. Those authors observed the cells were well adapted to the hydrogel scaffold culture condition and revealed important mechanisms of bacterial pathogenesis. Taking these examples together, it is clear that hydrogel scaffolds can be useful tools to investigate host-pathogen interactions of relevance for human health.

### 2.5 Organ-on-a-chip platforms

Organ-on-a-chip platforms are biomimetic tools that combine biomaterial technology, microfluidics, and tissue engineering to create miniature tissue devices, thereby allowing the recapitulation of the physiologically critical characteristics of specific human tissues and organs and their interactions on a small scale (29, 30). The chip is prepared with transparent and biocompatible polymers and contains continuously perfused chambers, where specific cell cultures can be deposited (29). Furthermore, the device has at least one channel with multiple interfaces (air-liquid, liquid-liquid), physical stimulation (mechanical shear or stretching forces), and mimics tissue architecture (29). Currently, organ-on-a-chip models have been developed addressing multiple organs, such as the gut, skin, kidney, lung, heart, liver, blood vessels, and bone marrow. Although there are still many challenges associated with these technologies, the innovative organ-on-a-chip approach is extremely useful for elucidating the pathophysiological characteristics of infectious processes caused by different bioagents in diverse body sites, bringing new insights into the immunological responses, as well as mimicking clinical responses to antimicrobial agents and spontaneous microbial evolution related to drug exposure (29, 69).

Over time, microengineering has advanced and evolved, allowing the use of this effective technology to build organ-specific microenvironments to reconstitute tissue structures, tissue-to-tissue interactions, and interfaces, enabling the study of dynamic mechanical and biochemical stimuli found in specific organs, and the targeting of cells to group them into suitable tissues (70).

Gut-on-a-chip models have proved to be powerful tools for studying commensal and intestinal pathogens. In a simplified way, the device development involves the design, manufacturing of parts, assembly, and examination, taking into account the anatomy of the designed organ to evaluate whether the flow speed and shear forces are in accordance with the physiological conditions of the human body (71). Kim et al. (72) created a biomimetic microdevice containing microfluidic channels separated by a flexible porous membrane, surrounded by an extracellular matrix, and lined by Caco-2 cells, imitating the human intestinal physiological structure. Additionally, conditions of fluid flow and peristalsis-like motions were created, allowing the development of a polarized epithelium, containing villi, and forming an integral barrier. Finally, the authors inoculated *Lactobacillus rhamnosus* GG, a probiotic strain, which was successfully cultivated without compromising epithelial protection, and improving the intestinal protective barrier (72). Gut-on-a-chip microfluidic devices have also been applied to study virus-host interactions, using the enteropathogen coxsackievirus B (CVB) (15, 73). Exposure of CVB to the apical surface of the epithelium led to successful viral replication, induction of cytopathic effects, and polarized (apical) release of pro-inflammatory cytokines (15, 73). Basal side infection led to a decrease in viral titers and lower cytopathic effects, with apical secretion of pro-inflammatory cytokines. Grassart et al. (74), used the intestine-chip technology to address *Shigella flexneri* intestinal colonization and demonstrated the invasion of enterocytes through the apical portion, leading to loss of mucosal integrity. Also, those authors observed that *S. flexneri* took advantage of the peristaltic-like movements to invade the epithelium, a feature which would not have been possible to replicate using conventional *in vitro* study models (74).

Organ-on-a-chip models have also been used to study other infection sites. For example, to evaluate host-*Mycobacterium tuberculosis* interactions, Thacker et al. (75) developed a lung microdevice containing alveolar epithelial cells, macrophages, microchannels to mimic air and blood flow, plus surfactants. Those authors observed that, in the presence of surfactants, *M. tuberculosis* grew very slowly, both in the lung and immune cells or even did not grow at all, depending on the culture conditions. The authors also reported that the growth of *M. tuberculosis* into macrophages was attenuated in the presence of lung surfactants due to the removal of virulence-associated lipids from the bacterial cell surface (75). Other organ-on-a-chip models have been proposed to evaluate aspects of intricate parasite-host relationships, such as a liver-chip to address hepatitis B virus infection (76), a spleen-chip to study *Plasmodium falciparu*m infection (77), an alveolus-chip model to evaluate co-infection with *S. aureus* and influenza virus (78), and an intestine chip to monitor ETEC infection (79), among others.

## 3 High-throughput sequencing methods

### 3.1 Transcriptomics and proteomics

Two of the major methods to get insights into the response of host cells to bacteria and, vice versa, are large-scale sequencing of RNA (transcriptomics or RNA-seq) and high-throughput identification of the protein content of the cells (proteomics). Transcriptomics has the benefit of being a relatively cheap “catch-all” type of method, in which the expression of all genes in a tissue can be quantified simultaneously and compared between conditions. In eukaryotic cells, one can take advantage of the poly-A-tail of mRNA to exclude most of the other forms of RNA (predominantly rRNA) contained in the cell, allowing to target the sequencing efforts towards protein-coding mRNA specifically. This has also enabled spatial transcriptomics (80, 81), in which transcription can be measured and connected to precise regions of the tissue, with close to individual eukaryotic cell resolution. However, this is not possible for bacteria, as they lack the poly-A-tail. This also means that in mixed samples, only a small fraction of the reads derived from an RNA-seq experiment will correspond to bacterial transcripts encoding proteins, requiring very high sequencing depths to perform relevant comparisons between conditions. Therefore, proteomic approaches might be more desirable in many cases to allow for studies of expressed proteins in both host and bacterial cells. Proteomic methods can also be targeted towards specific cellular compartments, such as the cell membranes or the cytoplasm, enabling an even stronger focus on the processes of interest.

### 3.2 Metagenomics and metatranscriptomics

When sequencing is performed on multi-species communities rather than individual species, it is referred to as metagenomics or metatranscriptomics (and correspondingly metaproteomics for proteins). Metagenomic sequencing of DNA has revolutionized our understanding of the diversity of bacteria and their functions, as it allows characterization of the uncultivable fraction of microbial communities. Analogously, metatranscriptomics and metaproteomics provide better pictures of the genes being expressed in a microbial community, or its protein content, respectively. This has been highly useful in deciphering a variety of host-associated environments, most predominantly the human gut (82–87), but also other body sites, such as the skin (88), stomach (89), vagina (90) and nasopharynx (91). It has also been successfully applied to better understand microbial ecosystems in a multitude of other environments (92–96). Yet, despite the versatility of these techniques, there are limitations to what can be achieved using them. A major hurdle is in the annotation of the sequence data. In principle, a good and representative reference data set is required to draw relevant conclusions based on the sequenced reads. Here, the most prominent issue is usually a lack of reference genomes to compare the reads to. Such lack can result in a large fraction of the obtained reads going unannotated (97, 98) creating large degrees of uncertainty. This uncertainty disproportionately will be associated with microbial species that are less well studied, biasing the analysis towards bacteria that are already well-characterized, such as *E. coli* and other Proteobacteria. While the magnitude of this problem has decreased as more genomes have been sequenced, this is still a major issue when undertaking metagenomic or metatranscriptomic sequencing in many environments, including the human body.

Another concern with the analysis of both transcriptomics and proteomics data is that the underlying assumptions of the analysis methods work well in single species studies, and to some degree in studies of host-single bacterium interactions. In the latter case, one major concern is the sometimes dramatically different abundances of host vs. bacterial material in various sample types. This problem can relatively easily be overcome by normalization methods when only one bacterial species is present together with the host. However, as the bacterial community combined with the host cells gets more complex, the problem with differential abundances gets exacerbated, as species in the microbial community may be present at vastly different abundances, sometimes differing by several orders of magnitude. This means that a transcriptomic or proteomic study in mixed-species communities may only be able to detect changes in transcription or protein composition for the most abundant microorganism(s) (99). Sometimes, the host material almost completely obscures the picture even in single-species studies. Two *in vivo* examples of these problems are studies of transcriptomes in the gastric environment (100) and the vagina (101). Both these environments are most often dominated by a single bacterium (*Helicobacter* in the stomach and different *Lactobacillus* species in the vagina) together with a vast amount of host cells. This means that while it is possible to detect other bacteria present in these environments, it is generally very hard to identify genes with differential expression in any other species than the dominant one. This also complicates comparisons between samples, particularly in the vaginal example where different species can be dominant in different persons.

Currently, the only feasible options to overcome these issues are to adapt the experimental methods or to use specialized normalization methods. When the microbial community composition is known, as in the case with microbial model communities (see section 4), target capture methods aimed to “fish” out the genomes of interest from a sample can be used (102, 103). One can also use cell sorting techniques to selectively perform, e.g., transcriptomics on only certain species in a community (104). This practice also allows measuring expression responses in single cells. Cell sorting is greatly eased using fluorescent markers, which allows the separation of different species in a microbial community (105). As host cells are usually much larger than microbial cells, these can typically be separated based on cell size. Regardless of the community, one can also apply more general approaches such as depletion of host DNA or RNA (106), depletion of rRNA in the case of metatranscriptomics (107), as well as applying combinations of metagenomics and metatranscriptomics that allow to better control for differences in gene expression levels between species (108).

Many of the problems with community transcriptomics (metatranscriptomics) can be overcome if one focuses on the DNA level instead of on expressed RNA. However, that comes with the loss of information on what genes are actually expressed in specific settings. The upside of this approach is that the differences in abundance between different species can be less extreme at the genomic (metagenome) level than at the transcriptional level, allowing for better detection of species that are enriched under certain conditions. Yet, if the abundance levels between species are extremely different, metagenomics suffers from the same issues as metatranscriptomics and metaproteomics. Overall, metagenomics, metatranscriptomics and metaproteomics are all formidable techniques to get better insights into host-associated communities. That said, they also all come with limitations which need to be taken into account when interpreting the outcomes of these experiments.

### 3.3 Large-scale transposon mutagenesis

**Figure 4:**
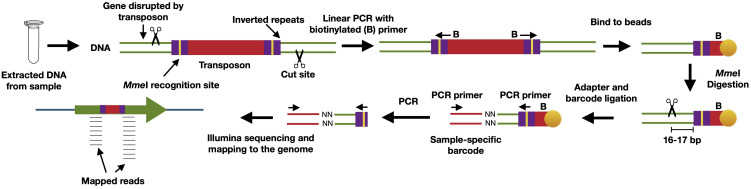
Overview of the INSeq protocol, used as an example of a transposon sequencing technique. In this protocol, PCR amplification of 16-17 bp of DNA flanking each disrupted gene allows the fitness of individual mutants to be calculated through DNA sequencing. Specifically, a recognition site for the restriction enzyme MmeI has been inserted into the inverted repeats of the transposon. By using a linear PCR with a biotinylated primer (B), followed by binding of the PCR products to streptavidin beads, MmeI can cleave the DNA flanking a disrupted gene upstream of the transposon, leaving 16-17 bp of the gene sequence attached to the bead. This can subsequently be used for a regular PCR step, by ligating another DNA sequence containing an Illumina sequencing adapter and the binding site for another PCR primer, which will selectively amplify the 16-17 bp regions of disrupted genes. These can then be sequenced and quantified, allowing the determination of fitness effects by specific gene knock-outs at a large scale.

Metagenomics can measure selective effects on the abundances of microorganisms, genes, and encoded functions in microbial communities, while metatranscriptomics and metaproteomics offer insights into the short-term responses to changing conditions. A complementary approach to these techniques is transposon mutagenesis. This term encompasses several specific protocols, all based on the same principles, aiming to characterize and quantify selection effects on individual genes at a very large scale (109). In brief, these protocols are based around a transposon – a piece of DNA that encodes a transposase protein that allows the piece of DNA to insert itself elsewhere in the genome – carried on a plasmid, which can be triggered to insert itself at a random location of a bacterial chromosome (110–112). Often the transposon used is a modified mariner transposon containing restriction enzyme recognition sites at its ends (**Figure 4**). The way these constructs are created allows for following individual viable deletion mutants using high-throughput sequencing, where specific tags associated with the gene disrupted by the transposon can be mapped back to the genome and identified. The benefit of these protocols is that both generation and detection of mutants can be achieved with very high throughput, in both *in vitro* and *in vivo* systems. By utilizing paired designs, in which the same source library of mutants is used for both a condition (such as growth in the presence of host cells) and a corresponding control (such as growth without host cells), the fitness for a given mutant can be directly compared between conditions. The paired setup allows for accurate and sensitive detection of mutants in genes important for growth under certain conditions, and specialized statistical packages exist for the analysis of such paired data (113).

Transposon mutagenesis and sequencing allow for the determination of the fitness effects of essentially every non-lethal gene deletion in bacterial genomes. It has been applied across a whole range of organisms from different phyla, including *E. coli* (114), *Klebsiella pneumoniae* (115), *P. aeruginosa* (116), *Bacteroides thetaiotaomicron* (117), as well as *Streptococcus* and *Staphylococcus* species (118, 119). While the molecular protocols required to transform the plasmid into bacteria from different taxonomic groups may differ substantially, the overall approach has shown remarkable versatility. It can also be used both *in vitro* and *in vivo* (114), again highlighting its flexibility as a large-scale tool for assessing fitness costs associated with specific conditions and genes.

In the specific context of host-bacterial interactions, transposon mutagenesis can be used to specifically determine which genes are involved in surface colonization (120), cell communication between bacteria and host (121), and overall fitness in the host environment (114, 117). In addition, these techniques have also been used to study specific genes involved in tolerance and resistance to antibiotics (122–124), as well as those involved in competition and virulence (125). To study interactions between bacteria and host cells at a large scale, one could design experiments in which transposon mutant libraries containing a mixed pool of tens of thousands of mutants defective in specific genes are added to pre-grown organoids under a variety of conditions. This allows the study of specific genes important for bacterial adhesion to tissues, growth in the presence of host cells, as well as colonization and invasion of organoid representatives of human organs. In addition, since these analyses can be performed with high throughput, these fitness effects can be probed across a range of conditions and concentrations, enabling the resolution of complex environment-host-microbe dependencies.

Notably, as with every other high-throughput sequencing-based technique, transposon mutagenesis will generate a high degree of false positive observations. Therefore, caution has to be taken to make sure that appropriate multiple testing correction is applied to the statistical data. This is usually possible in all statistical packages for this type of analysis (113, 126–128), but is sometimes not the default option and it is important to make sure to use these correction techniques as appropriate when dealing with this type of data. Furthermore, any potentially important gene should be retested in independent experiments with individual single-gene mutants. This practice ensures that any noise introduced from the other mutants in the pooled libraries can be disregarded. Finally, the transposon mutant studies are somewhat limited in specific types of mutant phenotypes for which they can detect significant effects. For example, a mutant deficient in some secreted ‘common goods’ molecule (such as a signaling molecule) would be able to hitchhike on the other mutants in the pool that are deficient in other genes but can produce the common goods molecule. Thus, transposon mutagenesis techniques are restricted to finding genes involved in individual cell fitness and are more likely to identify genes as important if they are, e.g., involved in direct cell-to-cell contact via the membrane, transport of certain molecules from the cell via efflux pumps or intracellular enzymatic processes involving molecules that cannot be easily taken up from the local environment.

## 4 Microbial model communities

Similar to how organoid systems can be cost-efficient, relatively easy-to-use models that can function as alternatives to animal experiments, but can yet provide a more realistic picture of the complexity of human organs than 2D cell cultures, microbial model communities are model systems aiming to capture interspecies interactions without the full complexity of real-world microbial communities encompassing hundreds or thousands of species (16). Microbial model communities – also sometimes referred to as synthetic communities (SynComs) – consist of a small number of microbial species and strains, chosen in a way that allows the study of interaction phenomena in a controlled manner. A range of microbial model communities exist, targeting different research questions, species combinations, and environments (16). In the context of 3D cell culturing approaches, it is of particular interest to mention those aiming to capture interaction phenomena among bacteria inhabiting humans. For example, the complexity of the human gut microbiota has been targeted in both 14-species (129) and 12-species (130) models, as well as in models aiming to capture a larger swath of the intestinal microbiota diversity (131, 132). These model communities contain different species, represent different degrees of complexity, and cater to somewhat different research needs. Notably, only three species overlap between the 14- and 12-species models, with the species in the model developed by Venturelli et al. (130) being selected based on how well they mirror the functional and phylogenetic diversity of the human gut, while Gutiérrez and Garrido (129) chose to include species based on their involvement in inulin metabolism. Both these communities are aiming to recapture different aspects of interspecies interactions in the human microbiota. In contrast, the more complex communities used by Spragge et al. (131) and Wuyts et al. (132) rather focus on being comprehensive and then systematically remove species from a large pool to better understand ecological interactions (131) or drug metabolism in the human microbiota (132).

Furthermore, there are also a few different microbial model communities developed as wound or tissue infection models (133–135). These contain just two to three species, and they typically involve the opportunistic pathogen *P. aeruginosa* as a prominent member. This bacterium is among the most common ones included in microbial model communities (16) and is also relatively easy to do transposon mutagenesis in (136), making it – together with *E. coli* – an excellently well-suited pathogen model to use in organoid infection models aiming to understand pathogenicity. There are also microbial model communities for the human lung (135) and stomach (Mirjam Dannborg et al., personal communication) environments, which would also be highly suitable to combine with organoid studies.

The benefit of introducing microbial model communities as a tool to better understand host-bacteria interactions is that they allow a degree of bacterial interplay to take place in addition to the direct host-cell and bacterial-cell interactions. This adds an additional layer of ecological complexity to these systems; for example, microbial communities may be protecting the human cells, providing colonization resistance against pathogens, as observed in the human gut (137, 138). There might also be situations where an opportunistic pathogen alone will not be able to significantly disturb the host organs, but where substances produced by other bacteria can induce a more virulent phenotype. Finally, competition between bacteria for, e.g., nutrients can introduce highly unpredictable behaviors towards host cells, which would be impossible to capture in single-microbe systems.

## 5 Combining large-scale -omics with 3D cell culturing approaches

A variety of pathogens have been studied using 3D models of enteroids, colloids and organoids, including *Salmonella* spp., *C. difficile*, enteropathogenic *E. coli* (EPEC), EHEC, ETEC, norovirus, rotavirus, enterovirus, *Toxoplasma gondii*, and coronavirus (15). By combining these infection model systems with large-scale -omics approaches, including transcriptomics, metagenomics, and transposon sequencing, we can gain both a deep and broad insight into how the pathogen and the host interact, and how the host tissue responds to pathogen invasion and vice versa. Notably, the combination of these approaches can also be used to study interactions between host cells and the beneficial microbiome in a semi-realistic setting. Here, the addition of microbial model communities makes it possible to also decipher the molecular and genetic mechanisms behind host-microbiome interactions. Below, we will highlight a few prominent examples of studies combining omics and 3D culture system approaches.

3D organoid models have been used to mimic human gastroenteritis shortly after *Salmonella* infection. The pathogen is here restricted to the epithelial compartment, which is an advantage over existing mouse models. Based on immunohistochemical and microscopic analysis, Nickerson et al. (57) reported that 3D cultured intestinal cells (Int-407) more accurately replicated the complex environment that *Salmonella enterica* serovar Thyphimurium faces during the natural course of human infection. The authors showed that there were differences in adherence, invasion, and tissue pathology between non-infected monolayers and 3D cultures. It is interesting to note that *S.* Thyphimurium presented low ability to adhere and invade 3D intestinal cells, which presented tissue organization and differentiation similar to *in vivo* conditions, for example, with regard to the presence of basement membrane proteins, microvilli, tight junctions, mucin and sialyl Lewis A (indicative of M-cell glycosylation). In contrast, intestinal Int-407 cells in 2D culture were poorly organized and presented reduced expression of cell differentiation markers. Although Nickerson et al. (57) did not employ nucleotide sequencing, it is one of the earliest examples of the use of large-scale techniques to investigate host-pathogen interactions in 3D models. The authors obtained total RNA from cultures to investigate the pattern of expression of cytokines by the intestinal cells following infection by *Salmonella*, based on a multiprobe commercial assay. It was shown that infection of Int-407 monolayers and 3D aggregates by *S.* Thyphimurium (1 to 2 hours) resulted in significantly higher expression of proinflammatory and immunomodulatory cytokines, including Tumor Necrosis Factor alpha (TNF-α) and several interleukins (IL-6, IL-1α, IL-1β), compared to uninfected monolayers and 3D culture. More specifically, TNF-α expression increased in monolayers after 1h and continued to rise after 2h, in contrast with 3D cells that presented TNF-α mRNA levels significantly elevated at 1h, but did not increase from 1h to 2h of infection. In fact, at 2 hours the TNF-α expression was more than fivefold higher in 2D cultures in comparison with 3D aggregates. Taken altogether, the results from Nickerson et al. (57) indicate 3D tissue aggregates from RWVs may overcome many limitations of monolayer assays for the investigation of bacterial infections.

Using dual host-bacteria transcriptome sequencing of a scaffold-based 3D model infected with *Salmonella*, Schulte et al. (53) revealed the communication between epithelial, endothelial, monocytic, and natural killer cells, but also with the pathogen. This study showed that downstream effects of infection on endothelial cells and immune cells not detected in monocultures could be captured in the 3D organoid model. In addition, the study showed that dual RNA-seq can identify bacterial virulence strategies, along with the responses of the infected host cells, highlighting complex time-dependent interactions of these systems that would be hard to capture in, e.g., a mouse model.

Bartfeld et al. (139) used organoids differentiated into specific lines of the stomach, presenting repetitive architecture with regular invaginations called gastric glands, to study pathogen-host interactions. These stomach organoids were used to investigate the response to *Helicobacter pylori* infection, measured as induction of cytokine mRNA (139, 140). The study combined image analysis with fluorescence-activated cell sorting (FACS), PCR and microarrays, allowing a multifaceted view on host-pathogen interactions. Organoids derived from different stomach tissues expressed their expected markers and assembled into gland and pit domains by self-organization. By adding nicotinamide and withdrawing WNT, the cell type of the organoids could be controlled, and the gastric gland lineages showed a strong inflammatory response to *H. pylori* (139).

Belanger et al. (141) used a combination of human skin organoid models and transposon sequencing in *P. aeruginosa* to identify genes important for survival during nosocomial infection. They found that genes typically upregulated during infection, such as those involved in transport of divalent cations and metallic cations, genes encoding the pyochelin synthesis proteins, as well as *pchG*, were indeed important in the organoid system, but not in typical lab media conditions. These genes also included those involved in nucleotide metabolism, iron uptake and vitamin B12 biosynthesis. This is consistent with previous studies of infection in *P. aeruginosa*, and highlights the usability of organoids as relevant host-infection models (141).

## 6 Future perspectives

The above examples outline some of the possibilities on the horizon with better model systems. With both organoid techniques, sequencing technologies – particularly more innovative applications of nucleotide sequencing – and microbial model communities rapidly developing into robust, large-scale, generally applicable tools, there are many reasons to expect a breakthrough at the intersection of these technologies. Particularly, there is a scarcity of research published using microbial model communities in combination with organoid models, utilizing large-scale -omics methods to understand the multiway interactions in these systems. While setting up such combination systems is complicated, the fact that there are several examples combining pathogens and organoids indicates that the time is ripe to bring together model communities and 3D culture systems. From there, using large-scale transcriptomics, metagenomics and transposon sequencing as read-outs for both the organoid tissue and the bacterial community responses seems like a natural step with few hurdles, aside from perhaps the additional costs.

One of the greatest difficulties currently encountered in this type of studies is the visualization of images, as the 3D structures are larger than monolayers due to the presence of several cells in different visual planes. Anchorage-dependent cultures and spheroids are also difficult to image due to plate incompatibility with microscopes (142). The most common way to analyze cellular phenotypes is using conventional wide-field fluorescence or confocal microscopy (142). In this area, new, faster methodologies must be developed, to overcome the current limitations faced by 3D cell culture.

Despite the need for further methods development in the imaging area, and the currently limited use of combinations of high-throughput molecular techniques, 3D culturing systems, and microbial model systems, this intersect provides a lot of promise towards reproducibility, consistency and scalability, which is simply not offered by animal models or *ex vivo* culturing techniques. Uniting these methods into a common framework will allow models that account for interspecies microbial interactions, host-microbe interactions, and tissue responses in a highly reproducible fashion, which also allows data to be extracted from the models at very large scales. This, in turn, will allow utilization of machine learning approaches to generate new hypotheses and to improve the design of future experiments. Ultimately, this will open up new avenues of research into microbial communities and their interactions with the human body, while at the same time reducing the need for animal experiments. Consequently, animal studies can then be targeted towards the most relevant and promising leads and phenomena identified in the model systems. Ultimately, the human body and the niches it offers microbial inhabitants are immensely complex biological systems. To understand this complexity, we need to reduce it into components where we can more easily control the parameter space, akin to how *E. coli* grown in the lab is used as a model system to understand bacteria and living cells in general. Based on these components, we can then figure out the larger picture in smaller steps, by extrapolating from simpler to increasingly more complex models.
